# Reply to “Unexpectedly higher mortality in non‑type 1 narcolepsy: diagnostic heterogeneity or unmeasured confounders?”

**DOI:** 10.1007/s44470-026-00120-9

**Published:** 2026-07-23

**Authors:** Amir Sharafkhaneh, Yves Dauvilliers, Ahmed S. BaHammam, Mehnaz Azarian, Michael Thorpy, Fang Han, Gulcin Benbir Senel, Murat Aksu, Javad Razjouyan

**Affiliations:** 1 Department of Medicine, Section of Pulmonary, Critical Care and Sleep Medicine, Baylor College of Medicine, 2002 Holcombe Blvd, Houston, TX 77030 USA; 2 Sleep Disorders and Research Center, Medical Care Line, Michael E. DeBakey VA Medical Center, Houston, TX USA; 3 Sleep-Wake Disorders Unit, Gui-de-Chauliac Hospital, CHU Montpellier, Institute of Neurosciences of Montpellier, University of Montpellier, Montpellier, France; 4 Department of Medicine, College of Medicine, University Sleep Disorders Center, King Saud University, Riyadh, Saudi Arabia; 5 Department of Medicine, Baylor College of Medicine, Houston, TX USA; 6 Center for Innovations in Quality, Effectiveness, and Safety, Michael E. DeBakey VA Medical Center, Houston, TX USA; 7 Albert Einstein College of Medicine, Bronx, NY USA; 8 Department of Sleep Medicine, Peking University People’s Hospital, Beijing, China; 9 Department of Neurology, Cerrahpasa Medical Faculty, Istanbul University-Cerrahpasa, Istanbul, Turkey; 10 Department of Neurology, Acibadem University, Istanbul, Turkey

**Keywords:** Mortality, Narcolepsy, Hypersomnia, Stimulant, Oxybate

We thank Dr. Fei and colleagues for their thoughtful commentary [1] on our recent publication [2]. Their letter offers helpful clarifications for readers regarding the nuances of using big data extracted from electronic health records. We intentionally defined the other narcolepsy (ON) group to represent a heterogeneous category that may include NT2, unspecified narcolepsy, and potentially some misclassified hypersomnolence conditions. This heterogeneity is an inherent characteristic of real-world electronic health record (EHR) phenotyping and is clearly acknowledged in our manuscript. Our design required ≥ 2 ICD codes, 30–390 days apart, to improve diagnostic specificity, minimizing transient miscoding and diagnostic uncertainty. We acknowledge the well-recognized limitation that Fei and colleagues raise regarding the multiple sleep latency test (MSLT), particularly the work of Trotti and colleagues [3] showing poor test–retest reliability in noncataplectic hypersomnia. Importantly, MSLT data were not available in a standardized manner across the national VHA dataset, and our cohort was constructed using a validated, computable phenotyping approach analogous to that applied by Lipford and colleagues at Mayo Clinic [4]. This algorithmic approach does not eliminate misclassification, but it minimizes single-code phenotyping errors [5, 6]. Because ON captures a mixture of clinical phenotypes with potentially some being undertreated, one may expect mortality rates equal to or higher than NT1. Our findings highlight this heterogeneity but do not imply that NT2 itself carries inherently higher mortality.

Our data showed an OSA prevalence substantially higher in GSC (65.4%) compared to the two narcolepsy groups (33–37%). Fei and colleagues suggested that PAP therapy may have lowered the mortality in the general sleep clinic (GSC) cohort, citing Benjafield and colleagues’ recent meta-analysis suggesting reduced all-cause mortality with PAP use [7]. While this is a possibility, it does not explain the mortality difference between ON and NT1. Thus, differential OSA prevalence does not account for the higher mortality observed in ON relative to NT1. Interestingly, the higher mortality in narcolepsy persisted despite the GSC cohort exhibiting a substantially higher burden of cardiovascular, metabolic, pulmonary, and psychiatric comorbidities at baseline. This suggests that the observed mortality differences are not simply due to comorbidity profiles. We emphasize in our manuscript that our study cannot identify causal mechanisms; instead, it highlights a significant association that warrants further investigation in cohorts with richer physiologic and treatment-adherence data.

In our real-world data, NT1 patients received sodium oxybate, stimulants, and wake-promoting agents more frequently than the ON group. This pattern likely reflects greater diagnostic certainty in NT1 and the established therapeutic indications for cataplexy, rather than disparities in clinical access. Fei and colleagues appropriately note that low-sodium oxybate is associated with a more favorable cardiovascular profile [8], an observation that supports the hypothesis that pharmacologic exposure may itself modulate mortality risk. We agree with Fei and colleagues that differential treatment exposure warrants further research, but this does not invalidate our findings; rather, it may represent an important mechanism contributing to poorer outcomes in ON.

Finally, we agree fully with Fei and colleagues that future studies integrating additional data sources, such as device-level PAP data or pharmacy fill trajectories, would be valuable for refining risk estimates. The broader literature on narcolepsy mortality remains mixed: a recent Taiwanese cohort by Hsu and colleagues reported no excess all-cause or cause-specific mortality after adjustment for comorbidities, [9] whereas our VA findings demonstrate elevated mortality relative to a clinically complex sleep-clinic comparator. Reconciling these observations will require harmonized phenotyping, cause-specific death ascertainment, and treatment-exposure modeling across diverse health systems. We hope our work, together with the constructive critique offered by Fei and colleagues, will encourage such efforts.

## Data Availability

Not applicable as this is a response to a letter submitted to JCSM.
